# The Canadian Bandaging Trial: Evidence-informed leg ulcer care and the effectiveness of two compression technologies

**DOI:** 10.1186/1472-6955-10-20

**Published:** 2011-10-13

**Authors:** Margaret B Harrison, Elizabeth G VanDenKerkhof, Wilma M Hopman, Ian D Graham, Meg E Carley, E Andrea Nelson

**Affiliations:** 1 School of Nursing, Queen's University, Kingston, Ontario, Canada; 2 Department of Anesthesiology, Queen's University, Kingston, Ontario, Canada; 3 Clinical Research Centre, Kingston General Hospital, Kingston, Ontario, Canada; 4 Department of Community Health and Epidemiology, Queen's University, Kingston, Ontario, Canada; 5 Canadian Institutes of Health Research, Ottawa, Ontario, Canada; 6 School of Nursing, University of Ottawa, Ottawa, Ontario, Canada; 7 School of Healthcare, University of Leeds, Leeds, UK

## Abstract

**Background:**

*Objective: *To determine the relative effectiveness of evidence-informed practice using two high compression systems: four-layer (4LB) and short-stretch bandaging (SSB) in community care of venous leg ulcers. *Design and Setting: *Pragmatic, multi-centre, parallel-group, open-label, randomized controlled trial conducted in 10 centres. Cognitively intact adults (≥18 years) referred for community care (home or clinic) with a venous ulceration measuring ≥0.7cm and present for ≥1 week, with an ankle brachial pressure index (ABPI) ≥0.8, without medication-controlled Diabetes Mellitus or a previous failure to improve with either system, were eligible to participate.

**Methods:**

Consenting individuals were randomly allocated (computer-generated blocked randomization schedule) to receive either 4LB or SSB following an evidence-informed protocol. Primary endpoint: time-to- healing of the reference ulcer. Secondary outcomes: recurrence rates, health-related quality of life (HRQL), pain, and expenditures.

**Results:**

424 individuals were randomized (4LB n = 215; SSB n = 209) and followed until their reference ulcer was healed (or maximum 30 months). An intent-to-treat analysis was conducted on all participants. Median time to ulcer healing in the 4LB group was 62 days [95% confidence interval (CI) 51 to 73], compared with 77 days (95% CI 63 to 91) in the SSB group. The unadjusted Kaplan-Meier curves revealed the difference in the distribution of cumulative healing times was not significantly different between group (log rank χ2 = 0.001, *P *= 0.98) nor ulcers recurrence (4LB, 10.1%; SSB, 13.3%; p = 0.345). Multivariable Cox Proportional Hazard Modeling also showed no significant between-bandage differences in healing time after controlling for significant covariates (p = 0.77). At 3-months post-baseline there were no differences in pain (no pain: 4LB, 22.7%; SSB, 26.7%; p = 0.335), or HRQL (SF-12 Mental Component Score: 4LB, 55.1; SSB, 55.8; p = 0.615; SF-12 Physical Component Score: 4LB, 39.0; SSB, 39.6; p = 0.675). The most common adverse events experienced by both groups included infection, skin breakdown and ulcer deterioration.

**Conclusions:**

The Canadian Bandaging Trial revealed that in the practice context of *trained RNs using an evidence-informed protocol*, the choice of bandage system (4LB and SSB) does not materially affect healing times, recurrence rates, HRQL, or pain. From a community practice perspective, this is positive news for patient-centred care allowing individual/family and practitioner choice in selecting compression technologies based on circumstances and context.

**Trial registration:**

clinicaltrials.gov Identifier: NCT00202267

## Background

Community care of individuals with chronic wounds has become an important issue for home care authorities as they deal with a growing demand for wound care in the community. In Canada, the impact of caring for individuals with leg ulcers is only now being recognized due to the increased pressure on the home care environment caused by nursing shortages and tightening of healthcare budgets [1]. In the UK, Luker and Kenrick estimated that up to 13% of community nurses' time was spent in leg ulcer care [2], and more recently NHS leg ulcer treatments (bandages and dressings) were reported to cost £300-600 million per annum in England [3]. Our previous work in one Ontario region demonstrated that 192 people with leg ulcers received on average 12 homecare visits per month, and expenditures for supplies and nursing visits were estimated at $1.3 million per annum [4]. While there are no national data, it is reasonable to believe that leg ulcer care in Canada consumes hundreds of millions of dollars annually.

For individuals with leg ulcers, the condition has a profound impact on quality of life [5-7]. It is chronic, may take years to heal, 66% of individuals experience recurring ulceration and 45% have a history dating back ten years [8]. Studies using the Short Form (36) Health Survey (SF-36^®^) instrument to measure health-related quality of life (HRQL) have demonstrated that individuals with leg ulcers have lower mean scores than Canadian population norms [9-11]. Multiple studies document the discomfort with venous leg ulcers, with the prevalence of pain estimated to be between 48-96 percent [12-23].

Best practice recommendations, supported by high level evidence, indicate that the most effective treatment for venous leg ulcers is high compression bandaging [24-36] applied by well-trained healthcare professionals [37-40]. However the initial Cochrane review had little evidence about the comparative effectiveness of the available types of high compression [41,42]. This is an issue given there are substantive clinical, practical and economic differences in the classes of compression technology. Early trials of bandaging systems were methodologically weak due to factors such as being underpowered [25]. By 2000 the VenUS I trial [43] was underway in the UK to determine the relative effectiveness of two of the most popular high compression systems, 'four-layer' (4LB: strictly speaking a four-component bandage system) and short-stretch bandaging (SSB). As the effectiveness and costs of the compression systems are likely related to service delivery and bandaging skills, we realized that the UK trial would not be able to completely answer the question for the Canadian healthcare system. In 2002, a Canadian team, working with the leads of VenUS I (Drs. E.A. Nelson and N. Cullum), set out to conduct a concurrent trial to provide more data about clinical and cost-effectiveness of the high compression technologies since the results of available published trials (Duby, Scriven, Partsch: n = 226 participants) were equivocal [44-46]. The Canadian trial, funded by the Canadian Institutes of Health Research (CIHR), was launched in 2004. Canadian homecare authorities were interested in a comparison of these commonly available bandaging systems based on a health services outcome, i.e. a 4-week difference in healing based on known costs of delivering the technologies in Canada. Thus, the purpose of this Canadian trial was to compare healing rates between two bandaging technologies, specifically: 1) determine if healing occurred at least 4 weeks earlier with SSB vs. 4LB; 2) assess related outcomes such recurrence rates, HRQL, pain, and adverse events over a one-year follow-up; and 3) identify potential baseline factors that predict healing.

## Methods

The Canadian Bandaging Trial (CBT) was a multi-centre parallel-group, randomized controlled trial conducted in 10 centres in three provinces. Recruitment involved all individuals referred for community wound care services (homecare or nurse clinic) with new, existing or recurrent venous ulceration. Ethics approval for the trial was received from Queen's University Research Ethics Board, Kingston Canada (REB# NURS-140-03).

### Study population

Individuals in community care were eligible for inclusion based on the following: adult (≥18 years), English-speaking or with access to translation, able to provide written informed consent, clinical presentation of venous insufficiency with an ankle brachial pressure index (ABPI) ≥0.8, and a leg ulcer with minimum duration of one week that measured at least 0.7 cm in any one dimension. After conducting a small pilot study, the eligibility criterion was changed from having an ulcer of at least 1 cm in any one dimension to 0.7 cm since it was found that too many individuals were being excluded that would have normally been treated with compression. Exclusion criteria were: medication-controlled diabetes mellitus, failure to improve over a 3-month period with either bandaging system prior to the trial, previous enrollment in the trial, and cognitive impairment.

#### Procedures

All participating study sites were supported to develop their evidence-informed protocols for venous leg ulcer management during the preliminary and pilot phase of this enquiry [47]. Protocol development is described elsewhere [10,48] but in brief, the local protocol was developed from guideline recommendations using a systematic 10-step adaptation process [49,50]. Training was provided on the use of both SSB and 4LB technologies and site investigators conducted random audits of protocol delivery to ensure ongoing compliance.

Upon referral for leg ulcer care, individuals received a comprehensive, standardized clinical assessment by specially trained registered nurses (RNs). Individuals were informed of the study and invited to participate. Consenting individuals were randomly allocated by research staff, available 7 days/week over day and evening hours via a remote telephone service, to receive one of two high compression technologies; 4LB or SSB. A computer-generated blocked randomization schedule was used to allocate participants. Allocation was sealed in opaque, serially numbered envelopes. Randomization was controlled centrally from the university research office and stratified by centre, ulcer size (≤ 5 or > 5 cm^2^), ulcer duration (≤ 6 or > 6 months), and whether they had a previous ulcer, as these factors may influence healing rates [51,52]

#### Intervention: High Compression Bandages

The 4LB system (control arm) was originally developed in the UK (Charing Cross Hospital) [53]. The commercial product widely used in Canada is Profore^® ^(Smith & Nephew Medical Ltd.). Precise components of the 4LB depend on the ankle circumference [54]. Bandages can remain in situ for up to one week (e.g. if minimal wound exudate) with bandages being changed when required. Bandages were discarded after each use.

Cotton short-stretch bandages (SSB) Comprilan^® ^(Beiersdorf-Jobst, Inc.) were applied using the modified Putter technique [55]. Varying widths of bandage, e.g., 8 cm, 10 cm and 12 cm, were used according to the width of the limb. A layer of orthopedic wool padding was applied beneath the bandage to distribute the compression evenly. Bandages were changed when required, as determined by the attending nurse. Participants washed and reused the short stretch wherever possible. For both 4LB and SSB, bandaging continued until the affected limb completely healed.

### Data Collection and Management

Baseline data collection began at the time of initial assessment through interview, clinical assessment and chart review. Socio-demographic and clinical assessment, and measurements of primary and secondary outcomes were completed at baseline. Monthly, the participant's reference ulcer (defined as the largest ulcer at randomization) was traced onto acetate to measure the wound surface. Adverse events and referrals to specialists were recorded. A Participant Satisfaction Survey was administered one month post-baseline or at healing (whichever came first) to capture patients' perspectives of their allocated bandage treatment and health services received. Economic data were also recorded and a report is in preparation. Study participants were followed until their reference leg ulcer was healed or for a maximum of 30 months until March 31, 2009. After healing, follow-up continued for one year to assess durability of healing.

Quality assurance procedures [56-58], included a detailed protocol manual for the site study teams and a log record to track the status of participants throughout the duration of the trial. Participants were assigned a unique identifying number used on study documentation to ensure confidentiality. Withdrawals from either arm of the study were monitored and reasons documented. Case records (10%) were randomly selected to assess data entry accuracy every 3 months.

### Primary and secondary outcomes

The primary endpoint was time (weeks) to complete healing of the reference ulcer. A healed ulcer was defined as fully epithelialized; no scab remaining, and no drainage. The point of healing was confirmed by: 1) serial tracing of the leg ulcer and 2) photographing the ulcer at the time of healing which was verified by a remote researcher, masked to allocation (EAN). Secondary outcomes included recurrence rates, HRQL, pain, and expenditures over a one-year follow-up. Adverse events related to the bandages were tracked.

### Sample size calculation

Our trial was adequately powered to detect whether the median healing time of SSBs is 4-weeks shorter than that of 4LBs. Sample size calculation was based on time-to-healing. Using local research on leg ulcer healing and service delivery [10,48], and the largest available trial at the time comparing 4LB and SSB (Partsch 2001, n = 112) [45] it was postulated that the median time to healing with 4LB would be 16 weeks. To determine the minimally important difference (MID) that would warrant changing from 4LB to SSB as first-line therapy, practitioners and homecare administrators were consulted. The consensus was that to opt for SSB over 4LB would be justified if no additional nursing visits were required. Local experience showed that during the first month of treatment with 4LB, on average 2.5 visits/week were required, followed by ~1.5 visits/week until healing occurred. For SSB, on average 3 visits/week were required for the first month, then 2 visits/week thereafter until healing. Assuming a median time-to-healing with 4LB of 16 weeks, an average 28 nursing visits would be required. Given the higher frequency of nursing visits with SSB, 28 nursing visits would occur over 12 weeks of care. Therefore, for SSB to be considered an attractive option for Canadian homecare regions, it would have to demonstrate a median time-to-healing of 12 weeks or less, thus a 4-week difference in healing was identified as the MID in time-to-healing.

With a median difference of 4-weeks in healing time (baseline, 16 weeks), 80% power, and a 5% level of significance, it was determined that 207 participants would be required in each arm (414 total) allowing a 5% loss to follow-up. A review of our previous studies' recruitment and activity status data revealed that the lost to follow-up rate was slightly higher than 5% therefore the sample size was increased to 424 participants.

### Analysis

The primary analysis was based on 'intention-to-treat'. Index scores were generated for the Short Form McGill Pain Questionnaire (SF-MPQ), and SF-12^® ^Physical Component (PCS) and Mental Component Summaries (MCS) were derived using the Quality Metric SF Health Outcomes™ Scoring algorithm [59,60]. Individual missing items for the SF-12^® ^were imputed using assignment of mean score (AMS) [61,62]. Following a descriptive analysis, the baseline and clinical characteristics of the 4LB and SSB groups were compared. The primary outcome, time-to-healing of the reference ulcer, was compared between individuals randomized into the two groups. Kaplan-Meier survival curves were constructed for the two groups. The statistical significance of the difference between the two bandaging groups was tested using log rank tests. Cox proportional hazards modeling was used to identify baseline demographic and prognostic indicators of time to healing, in addition to assessing the effect of treatment group. Indicators included geographic location, sex, age, living situation, co-morbidities, pain, mobility, ankle flexion, edema, ABPI, ulcer episode, ulcer duration, and ulcer size [51,63-67]. Factors that predicted healing in bivariable analyses (p ≤ 0.15) were included in the multivariable analysis. The assumption of proportionality of hazards was tested for all factors using log-minus-log plots. The chi-square test was used to compare the proportion of ulcers that recurred in the two groups. Adverse events and participants' perceptions of the bandaging systems were compared using chi-square tests. All analyses were completed using IBM^© ^SPSS^© ^Statistics software (Version 19 for Windows).

## Results

**Figure 1:**
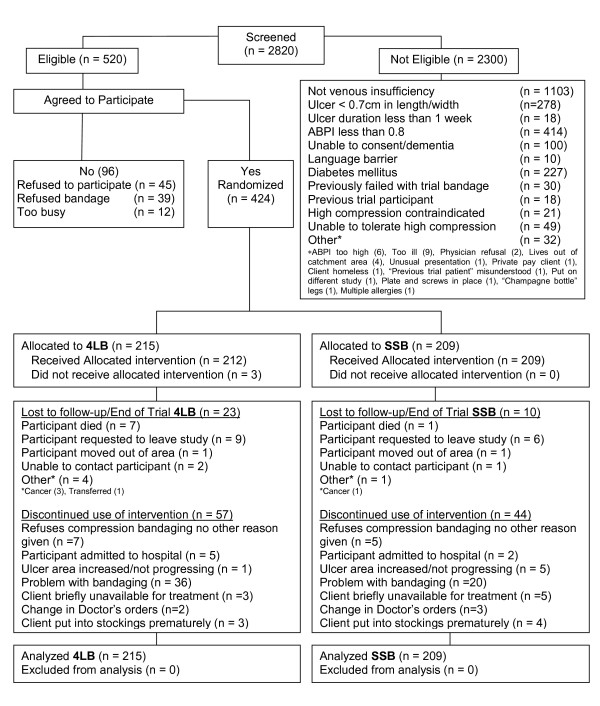
**CBT Recruitment and Randomization**.

Over a 50 month recruitment period (January 2004 - March 2008), 2820 individuals referred for community leg ulcer care were screened (see Figure 1). Many of those referred for care did not have purely venous leg ulcers, thus the trial procedures required a major screening effort. For example more than half screened had diabetic or arterial issues (hence mixed ulcer aetiology) but there was no way of knowing this prior to examination by the nurse and it was vital to maintain an evidence-informed clinical assessment consistently. Consequently only 520 were assessed as eligible with 82% of those (n = 424) agreeing to participate.

Of the 424 trial participants, 215 were randomized to receive 4LB, and 209 to receive SSB. There were no differences on admission between the participants randomized to either bandage on their socio-demographic, circumstance of living, HRQL or clinical characteristics (Table 1).

**Table 1:** Baseline and clinical characteristics of the study population by intervention group

Characteristics^1^	4LB (n = 215)	SSB (n = 209)	p-value
Gender (female)	119 (55.3)	111 (53.1)	0.70
Language (English)	189 (87.9)	195 (93.3)	0.16
Lives with others	140 (65.1)	131 (62.7)	0.61
Fully mobile	174 (80.9)	163 (78.0)	0.47
Non-venous history	125 (58.1)	132 (63.2)	0.32
Leg ulcer pain on admission^2^	183 (89.7)	182 (89.2)	1.00
Medications for leg ulcer pain	53 (24.7)	62 (29.7)	0.28
# Co-morbidities			
None	40 (18.6)	34 (16.3)	0.67
1 - 2	136 (63.3)	131 (62.7)	
≥ 3	39(18.1)	44 (21.1)	
Previous leg ulcers	93 (43.3)	98 (46.9)	0.50
Previous compression^3^	125 (58.4)	128 (61.2)	0.62
Reference ulcer on left leg	108 (50.2)	105 (50.2)	1.00
Edema on affected leg^4^	180 (84.9)	175 (84.1)	0.89
Full flexion on affected leg^5^	167 (79.9)	168 (82.0)	0.62
Age (years)*	64.4 (16.2) [23.8-93.0]	65.7 (17.0) [22.8-94.1]	0.41
Duration of current ulcer (weeks)†	11.7 (0.14-521.1)	11.4 (0.86-1044.7)	0.80^6^
Area (cm^2^) - Tracing^7^†	3.0 (0.16-139.8)	3.3 (0.07-50.8)	0.74^6^
ABPI on affected leg^8^*	1.05 (0.15) [0.79-1.7]	1.04 (0.15) [0.79-1.5]	0.60
McGill Pain^2^*			
Sensory Pain Index	18.5 (18.1)	19.5 (17.4)	0.58
Affective Pain Index	6.7 (14.7)	7.5 (15.9)	0.61
Total Pain Index	15.3 (16.0)	16.3 (15.8)	0.56
PPI-VAS^9^	2.5 (2.3)	2.8 (2.5)	0.24
SF-12^10^*			
Physical Component (PCS)	39.5 (10.5)	38.7 (9.2)	0.40
Mental Component (MCS)	51.9 (9.9)	50.9 (9.9)	0.34

^1 ^Values in parentheses are percentages unless indicated otherwise; *values are mean (s.d.) [range]; †values are median (range). 4LB, four-layer bandages; SSB, short-stretch bandages.

^2 ^Pain data were available for 204 participants in each treatment arm

^3 ^Previous compression data were available for 214 participants in the 4LB group

^4 ^Data on edema and dependency edema were available for 212 participants in the 4LB group and 208 in the SSB group

^5 ^Data on ankle flexion were available for 209 participants in the 4LB group and 205 in the SSB group

^6 ^Mann-Whitney U

^7 ^Ulcer tracing data were available for 206 participants in each treatment arm

^8 ^ABPI data were available for 214 participants in the 4LB group

^9 ^PPI-VAS; Present pain intensity - visual analogue scale

^10 ^SF-12 data were available for 204 participants in the 4 LB group and 203 in the SSB group. Values imputed using mean for individual missing responses

Mean age of the participants was 65 years with slightly more women (54%) than men and the majority being English-speaking (91%). Most participants were fully mobile and lived with others. For more than half (55%), this was their first episode of ulceration and the median ulcer duration at baseline was nearly 12 weeks. PCS baseline scores were much lower than the age- and sex-adjusted Canadian norms (39.1 vs. 51.7); indicating that those with venous leg ulcer had much poorer physically oriented HRQL. However, mental HRQL was better, with MCS scores comparable to Canadian norms (51.4 vs. 50.5) [68]. There were no differences in key aspects of the clinical care received by the two groups (Table 2).

**Table 2:** Adherence to the Evidence-Informed Protocol

*Variables*	Four Layer (n = 215)	Short-Stretch (n = 209)
	% (n)	% (n)
Comprehensive Clinical Assessment	100 (215)	100 (209)
Doppler ABPI	100 (215)	100 (209)
High Compression Therapy		
All	98.6 (212)	100.0 (209)
Venous disease	99.1 (210)	97.1 (203)
Mixed Disease	0.9 (2)	2.9 (6)

### Time-to-Healing

**Figure 2:**
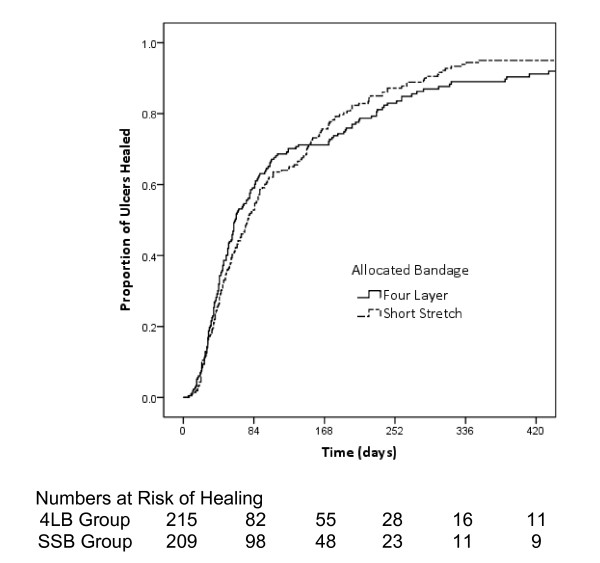
**Kaplan-Meier curves showing proportion of ulcers healed by treatment with four-layer and short-stretch bandages**.

The median time to reference ulcer healing in the 4LB group was 62 days [95% confidence interval (CI) 51 to 73], compared with 77 days (95% CI 63 to 91) in the SSB group. Figure 2 shows the unadjusted Kaplan-Meier curves. The difference in the distribution of cumulative healing times between the two groups was not significantly different (log rank χ2 = 0.001, *P *= 0.98).

#### Factors affecting time-to-healing

Several factors were associated with time-to-healing in bivariable analysis, however in multivariable analysis, only age, ulcer size at baseline and geographic location, predicted time-to-healing (Table 3).

**Table 3:** Treatment effect on time to healing adjusted for explanatory co-variates

		Bivariate Analysis		Multivariate Analysis	
	**Variable**	**Sig**.	**Hazard Ratio (C.I)**	**Sig**.	**Hazard Ratio (C.I)**

Centre^1^	Grouping 1	< 0.01	1.0	< 0.01	1.0
	Grouping 2	< 0.01	2.2 (1.6-3.1)	< 0.01	1.8 (1.2-2.6)
	Grouping 3	< 0.01	2.8 (2.0-4.0)	< 0.01	2.3 (1.6-3.4)
	Grouping 4	< 0.01	2.6 (1.8-3.7)	< 0.01	2.5 (1.7-3.7)
	Grouping 5	< 0.01	4.2 (3.2-5.6)	< 0.01	3.7 (2.7-5.0)

Gender^2^	Male	-	1.0	-	1.0
	Female	0.34	1.1 (.90-1.4)	0.26	1.1 (.91-1.4)

Age	Decades	< 0.01	1.1 (1.1-1.2)	0.04	1.1 (1.0-1.1)

Living situation	Alone	-	1.0	-	1.0
	With others	0.09	1.2 (.97-1.5)	0.08	1.2 (.98-1.5)

Co-morbidities	None	< 0.01	1.0		n.s.
	1-2	0.06	1.3 (1.0-1.7)		
	> 3	< 0.01	2.2 (1.5-1.3)		

Pain on Admission	Yes	-	1.0		n.s
	No	0.02	1.5 (1.1-2.1)		

Mobility	Fully mobile	-	1.0		n.s
	Walks with assistance/immobile	0.02	1.4 (1.1-1.7)		

Ankle flexion	Full flexion	-	1.0		n.s
	Impaired/no flexion	0.10	1.3 (.96-1.6)		

Edema	Yes	-	1.0		N/A
	No	0.86	1.0 (.77-1.4)		

ABPI		< 0.01	5.7 (3.0-10.9)		n.s

Previous Ulcer	Yes	-	1.0		N/A
	No	0.64	1.0 (.86-1.3)		

Ulcer duration	≤ 12 weeks	< 0.01	1.8 (1.5-2.2)		n.s
	> 12 weeks	-	1.0		

Ulcer size	≤ 2.5 cm	< 0.01	2.7 (2.1-3.5)	< 0.01	2.1 (1.5-2.7)
	> 2.5 to ≤ 10 cm	< 0.01	1.6 (1.2-2.0)	< 0.01	1.5 (1.1-2.0)
	> 10 cm	< 0.01	1.0	< 0.01	1.0

Allocated Bandage	Four layer	-	1.0	0.77	1.0
	Short stretch	0.99	1.0 (.82-1.2)		1.0 (.84-1.3)

^1^Study centres have been concealed for anonymity and grouped by geographic region where n < 50

^2^Gender and Allocated Bandage were forced into the model

^3^For the bivariate analysis, the sample size was 424 for most variables (408 for pain on admission, 420 for edema, 423 for ABPI) and 39 cases were censored. For the multivariable model, a total of 24 were excluded due to missing data and 36 were censored.

N/A = Variable not entered into Cox Regression (p > 0.15)

n.s. = Variable not selected for Cox Regression final model

### Secondary Outcomes

The proportion of people who experienced a recurrence of their reference ulcer after healing during the one year follow-up was relatively small (10% for 4LB, 13% for SSB; no statistically significant difference). At 3-months post-baseline there were no differences in pain (no pain: 4LB, 22.7%; SSB, 26.7%; p = 0.335) or HRQL outcomes (SF-12 MCS: 4LB, 55.1; SSB, 55.8; p = 0.615; SF-12 PCS: 4LB, 39.0; SSB, 39.6; p = 0.675) and little improvement from the group's low baseline values on the PCS.

### Adverse Events

Adverse events were tracked up to 30 months. In both groups the most common adverse events were infection, skin breakdown and ulcer deterioration with 29% of the 4LB group and 33% of the SSB group experiencing an adverse event (not significant, Table 4). The SSB group took 2 weeks longer to heal thus, were 'at risk' of adverse event occurrence for a longer period of time.

**Table 4:** Adverse events potentially related to compression bandages (max. 30 months)

	4LB (n = 215)	SSB (n = 209)	p-value
	n (%)	n (%)	
Pressure damage	15 (7.0)	13 (6.2)	0.85
Skin breakdown	27 (12.6)	34 (16.3)	0.33
Ulcer deterioration	27 (12.6)	32 (15.3)	0.48
Infection	28 (13.0)	35 (16.7)	0.34
New ulcer	13 (6.0)	22 (10.5)	0.11
Allergy/dermatitis related to bandaging	15 (7.0)	14 (6.7)	1.00
Allergy/dermatitis related to ointment/cream	9 (4.2)	10 (4.8)	0.82
Recurrence	2 (0.9)	5 (2.4)	0.28
Limb compromise	2 (0.9)	2 (1.0)	1.00

Total	138 (64.2)	167 (80.0)	

### Participant Satisfaction Survey

Analysis of participants' perceptions revealed 41% of the 4LB group reported experiencing a problem with their bandaging compared to 28% of those allocated SSB (p = 0.01). In particular, there was significantly greater discomfort with the 4LB (p = 0.05), and participants felt it was often applied to tight (p < 0.01). The majority in both groups reported being very satisfied with the nurses' skills in applying the bandage, and reported that the nurses provided verbal and written information regarding ulcer care and prevention (Table 5).

**Table 5:** Participant's perceptions at 1-month post-baseline of compression bandages and care received

	Four-Layer (n = 196)	Short Stretch (n = 199)	p-value
	n (%)	n (%)	
Reported problems with bandaging	81 (41.3)	56 (28.1)	0.01
Skin reactions	16 (8.2)	9 (4.5)	0.15
Discomfort	49 (25.0)	33 (16.6)	0.05
Skin breakdown	12 (6.1)	10 (5.0)	0.67
Applied too tight	35 (17.9)	10 (5.0)	< 0.01
Applied too loose	6 (3.1)	7 (3.5)	1.00
Satisfaction with nurses' skills applying the bandage^1^			
Very satisfied	154 (79.0)	171 (85.9)	0.10
Quite satisfied	40 (20.5)	25.0 (12.6)	
Neither satisfied or dissatisfied	1 (0.5)	1 (0.5)	
Quite dissatisfied	0 (0.0)	2 (1.0)	
Bandage comfort^2^			
I have to take them off because they are too uncomfortable	13 (6.7)	8 (4.0)	0.18
I wear them but they are very uncomfortable	6 (3.1)	2 (1.0)	
I wear them but they are slightly uncomfortable	47 (24.1)	41 (20.6)	
I have no trouble wearing them	129 (66.2)	148 (74.4)	
The nurse gave information about how to care for leg ulcer^3^	180 (92.3)	178 (89.4)	0.38
The nurse talked about preventing the ulcers from coming back^4^	161 (82.1)	164 (82.8)	0.90
The nurse gave written information about preventing the ulcers from coming back^5^	114 (58.2)	113 (57.7)	1.00

^1^Perception of nurses' skill applying bandage data were available for 195 participants in the 4LB group

^2^Bandage comfort data were available for 195 participants in the 4LB group

^3 ^Information given to care for their leg ulcer data were available for 195 participants in the 4LB group

^4^Verbal information given to prevent ulcer recurrence data were available for 198 participants in the SSB group

^5 ^Written information given to prevent ulcer recurrence data were available for 196 participants in the SSB group

## Discussion

The Canadian Bandaging Trial was adequately powered to detect a minimally important difference of 4-weeks in median healing time between 4LBs and SSBs. We put much emphasis on testing the compression technologies within an evidence-informed approach to care i.e. a protocol for venous leg ulcer assessment and management, well developed training procedures, and well prepared RNs. With these elements in place, results from our trial suggest that the two compression bandaging technologies tested are not different in terms of time-to-healing, recurrence rates, HRQL, or pain. However, individuals were less satisfied with 4LB, with more reporting problems with the bandaging system.

These healing results should be viewed in the context of all the trials making this comparison. O'Meara et al. [33] conducted a meta-analysis of individual patient data to evaluate the intervention effect of 4LBs relative to SSBs. They concluded that venous leg ulcers in patients treated with 4LBs heal faster, on average, than those treated with SSBs; and the benefits were consistent across patients with differing prognostic profiles. In contrast, our trial (the largest to date), put much emphasis on other aspects of venous leg ulcer care, namely an evidence-informed protocol of care and well-prepared RNs in administering these bandaging systems. With these elements of care in place, there were no differences between the technologies except that individuals may have more problems with 4LBs.

In the O'Meara meta-analysis, the intervention effect estimate from the UK VenUS I was weighted heavily towards the pooled effect estimate (i.e. 63% from this trial among five others). As the only other large scale trial comparable to this Canadian RCT, the UK VenUS I showed that with 4LBs there was a shorter healing time. We were unable to replicate this finding despite comparable sample sizes. Several factors may be contributing to this difference. Nurse wound-care teams in Canada have more training and experience in *both *technologies. The SSB was newly introduced to UK primary care just prior to their trial and there was a higher withdrawal rate in the SSB arm of the VenUS I trial. The UK authors [43] state they "investigated the *introduction *(their emphasis) of a high-level compression bandage system into a setting where an acceptable mode of compression was already in use" and a reasonable conclusion is that short stretch is "a useful alternative" (pg. 1298). Based on the experience from the UK trial, our Canadian sites had initial and ongoing training including quality checks to ensure adherence to the evidence-informed assessment and management protocol for either bandage. In the Canadian trial, the nurses may have been more confident and competent in delivery of both bandage systems thus reducing operator experience/preference as a source of bias. Another context factor may be important. Canadian site teams actively participated in the development of the protocol in their local settings to make it as seamless as possible within their normal delivery of care. Based on best practice recommendations, they carefully constructed an approach to adhere to key aspects of leg ulcer assessment and management. For example, the initial comprehensive assessment may have been carried out by specially trained nurses in the home with portable equipment whereas at some sites, it was more feasible at a designated clinic location where a wound expert and equipment were centralized.

In considering the site differences our team thought there were factors that should be further explored in a future study. Although we tracked key elements of the evidence-informed protocol (comprehensive assessment and use of compression), and all sites delivered these recommendations, there may be subtle differences contributing to healing rates. For example, some sites were newer and less experienced and may have been less supported regionally in the delivery of care. As well, because the trial was conducted in a large number of jurisdictions the referral and wait-times may have varied influencing the time to assessment which may have affected outcomes. Unfortunately we did not have data to drill down and explore these issues in this trial.

Lastly, healing in the Canadian trial for 4LB was 62 days compared to 77 days with SSB - both much shorter than the UK study population. This may be partly explained by their healing outcome being complete healing of all ulcers not just a reference ulcer. Nevertheless in examining healing over time compared to previous trials (Table 6), the Canadian group had higher proportions healed at 12, 16, 24 and 52 week time-points for both SSB and 4LB except SSB at 16 weeks in the Partsch study [45].

**Table 6:** Healing Rates at 12, 16, 24, and 52 weeks in trials comparing four-layer and short-stretch bandages

		Proportion of Ulcers Healed (%)							
		12 Weeks		16 Weeks		24 Weeks		52 Weeks	
Reference	Year	4LB	SSB	4LB	SSB	4LB	SSB	4LB	SSB
Duby et al. *	1993	44	40	-	-	-	-	-	-
Scriven et al. *	1998	34	41	-	-	-	-	53	56
Partsch et al. †	2001	-	-	62	73	-	-	-	-
Ukat et al.	2003	30	22	-	-	-	-	-	-
VenUS I†	2004	46	37	55	45	68	55	78	72
Franks et al. †	2004	-	-	-	-	69	73	-	-
Harrison et al.	2011	58	53	67	63	69	75	83	92

*In these trials the legs of several patients with bilateral ulceration were randomized independently. †Primary outcome in these trials was complete healing of all ulcers on trial leg (in the presence of multiple ulcers this would tend to underestimate the outcome of ulcer healing). 4LB, four-layer bandages; SSB, short-stretch bandages.

From this large Canadian trial the key message regarding healing is 'when delivered in the practice context of *trained RNs and an evidence-informed protocol *choice of bandage system (4LB and SSB) does not materially affect healing times, recurrence rates, HRQL, or pain'. Expenditures and resource use should be another consideration. According to results from the UK VenUS I trial on average, 4LB was associated with greater health benefits and lower costs than SSB. In a separate cost-effectiveness analysis, we are addressing whether this finding holds in the community care setting in Canada.

### Limitations of the Study

This trial has some limitations that should be considered when interpreting the results. It might be considered a limitation that nurses providing care were involved in collection of outcomes data. Blinding the nurse to the compression technology was not feasible and once bandages were applied it would have been excessively intrusive (and monumentally expensive) to remove them solely for the purpose of an outcome assessment. However, our small team of dedicated specially trained nurses, ensured expert, quality outcome assessments by measuring healing in a rigorous and consistent manner and this was validated by photo assessment by an expert remote from the site of care and who was blinded to allocation group. The results may not be generalizable beyond the study population but since the trial took place in multiple sites in three provinces (Ontario, Manitoba and Saskatchewan) that included both urban and rural locations, and culturally mixed populations this should increase the likelihood of generalizability.

### Strengths

This RCT is the largest community wound trial conducted in Canada and the largest trial evaluating these two compression technologies internationally. The trial's pragmatic approach emulated the usual delivery of assessment and care with healing outcomes confirmed by an individual blinded to study arm. The comparative effectiveness of SSB and 4LB systems in healing ulcers will be more precisely addressed when the Canadian trial is added to the meta-analysis from the Cochrane Wounds Group [33] recognizing that context is important and contributing to the sub-groups analysis of trial result by country of study.

## Conclusions

Internationally this is the largest reported trial on bandaging systems and should immediately contribute to body of evidence at the Cochrane Library and elsewhere. We were unable to replicate the results of the largest UK trial, VenUS I. Within the Canadian context, the implications are that homecare authorities and community providers can expect similar healing, quality of life, pain and recurrence results from 4LB and SSB compression technologies. It is vital to note that these results are dependent on compression bandages being delivered within an overall approach of evidence-informed care by well-prepared providers - *this cannot be overstated*. From a community practice perspective, these results are positive news as it allows individual/family and practitioner choice in selecting a technology class based on the circumstances and context. This means being better able to deliver patient-centred care. Given either bandage is effective, the choice can be made with individuals and practitioners based on bandage preference. The bandage systems require different self-care ability and supports, levels of nursing time, variation in individual comfort, and preparation and teaching. From a policy perspective, the organization of care can be adjusted to account for the different requirements with either bandaging technology. If delivery of only one, or the other, is possible because of local factors and resources, it is important to know that the expectation of healing would not be compromised whichever is selected. Several next steps to advance leg ulcer care emerge from this Canadian study, including:

• Conducting a meta-analysis to include this large Canadian trial to the existing reports on SSBs and 4LBs to increase power and add another country's experience

• Analyzing the economic implications of the Canadian trial for SSB and 4LB in order to position the effectiveness results for planners and policy makers.

• Addressing the deficit in evidence for people with diabetes who have leg ulcers. Although typically excluded from compression trials, in practice compression is used with this group. Healing rates and other outcomes are unknown (people with diabetes comprised ~24% of those excluded in this trial). We suggest the conduct of a pragmatic trial using a similar approach (qualified team, evidence-informed protocol).

• Research to integrate the multiple possible wound outcomes into administrative databases is needed to reduce the need for primary data collection for costly follow-up in large scale studies. In particular recurrence rates are clinically and administratively important but add enormously to trial costs. Issues include the organization, accessibility and quality of data currently collected in the community and home care environment. Chronic wounds e.g. leg ulcers (arterial and venous), diabetic and pressure ulcers, are increasingly being cared for in the community. Future wound studies would *all *be aided by improved administrative tracking of outcomes.

• Given the self-management aspects with compression bandages, research into the supportive care needed for self-management is an important area for enquiry.

In conclusion the Canadian bandaging trial adds new information to the international literature focused on the management of leg ulcers in the community. Two commonly available technologies, short-stretch and four-layer bandages, are viable options that result in similar healing, recurrence, HRQL and pain outcomes when delivered by trained nurses using an evidence-informed protocol.

## Declaration of Competing interests

The authors declare that they have no competing interests.

## Authors' contributions

MBH was principal investigator on the trial and responsible for conceptualization, ethical approval, the conduct and management of the RCT, analysis and interpretation of the data. IDG, EAN, and EGV were trial co-investigators and contributed to the conceptualization, analysis and interpretation of the data.

WMH was responsible for the HRQL analysis and contributed to interpretation of the trial data. MEC was responsible for data management and contributed to the analysis.

The 'Canadian Bandaging Trial Group' members served as site investigators and contributed to the feasibility and conduct of the trial protocol.

All primary authors have read and approved the final manuscript.

## Other information

### Registration

This trial is registered at http://www.clinicaltrials.gov, identified by number NCT00202267.

### Protocol

The full trial protocol is not publicly available at this time.

## Pre-publication history

The pre-publication history for this paper can be accessed here:

http://www.biomedcentral.com/1472-6955/10/20/prepub
